# Spreader Graft Easy and Stable Fixation

**DOI:** 10.1097/GOX.0000000000002168

**Published:** 2019-03-20

**Authors:** Héctor César Durán-Vega, Arturo Regalado-Briz

**Affiliations:** From the * Private Practice in Mérida, Yucatán, México; † Private Practice in Monterrey, Nuevo León, México.

## Abstract

The original spreader graft described by Sheen was dissecting a sub perichondrial flap along the anterior edge of the septum and inserting the grafts in place and occasionally, fixing them with a mattress suture. Although it has been done usually with mattress suture, it has some disadvantages as the instability of the graft while it is being fixated and others. We present a different fixation method with a simple running suture instead.

## BACKGROUND

Sheen described the spreader grafts in 1984^1^ as a new method to correct the middle nasal vault problems, especially in narrow nose syndrome or short nasal bones in primary rhinoplasty, and in secondary ones with a collapse in middle nasal vault because of an excess of resection (inverted V). His resolution was the spreader grafts, a couple of stick-like grafts placed along the dorsal edge of the septal cartilage. He dissected a subperichondrial flap along the anterior edge of the septum and inserted the grafts in place and occasionally, fixing them with a mattress suture.

Although open rhinoplasty became the first option for many surgeons, the fixation with a mattress suture has become usual. But fixation has been recognized in many publications as difficult or complex.^2–4^ The most frequent problems related, especially if the grafts are not fixated, are graft displacement, shearing, asymmetry,^3^ increased time,^5^ or to drop the graft into the mucoperichondrial flap.^6^ So authors have reported different fixation methods, for example, suturing the grafts as a saddle bag,^2^ with cyanoacrylate,^7^ modifications to the mattress suture,^8^ fibrin sealant,^9^ tight submucoperichondrial pockets,^10^ modified speculums,^11^ transcutaneous sutures,^5^ or barbed sutures.^12^

Usually, spreader grafts are obtained after septum mucoperichondrial dissection, and then fixed temporarily by using 27–30 gauge needles ^k^. Then a couple of horizontal mattress sutures are placed, so the previous complications mentioned before are not so common. But we find it difficult to perform it because of the lack of short straight needles in hospitals, movement of the grafts while passing the needle, lack of symmetry, or displacement of the spreader graft alignment or instability.

## SURGICAL PROCEDURE

**Fig. 1.**
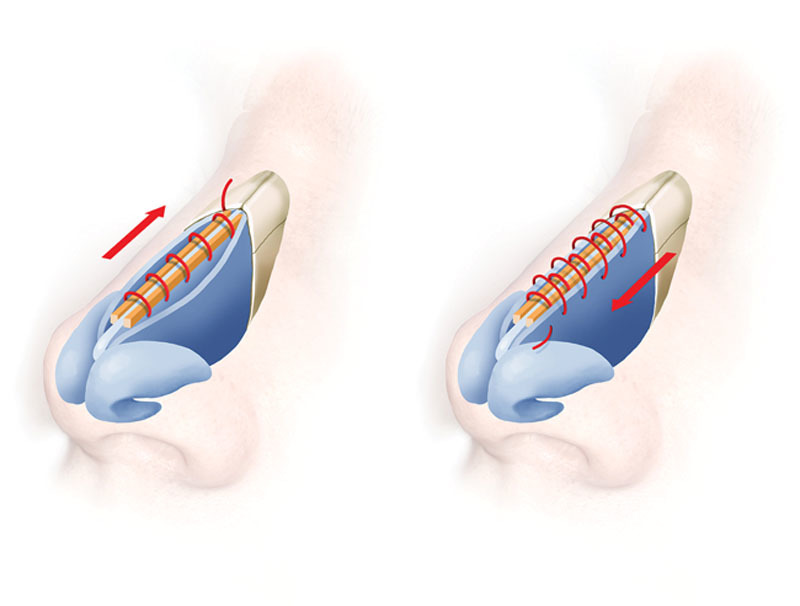
Running suture from caudal to cephalic embracing both spreaders at the side of the septum and coming back from cephalic to caudal fixating also the upper lateral cartilages.

We did all the rhinoplasties with an open technique, in primary or secondary cases during a 5-year period (2013–2018). Using 4-0 nylon (or an absorbable one) with a short, round, curved needle, we start with a simple suture embracing both grafts and septum in the most caudal portion of the septum; sometimes it can be done with 5-0. Then without cutting the tail (it will be used at the end), continue as a simple running suture directed cephalically along the dorsum. The needle penetrates the septum a millimeter away of the spreader width so each time it runs embraces both spreader graft and septum while accomplishing these objectives: fixation, alignment, symmetry, and stability. Sometimes while doing initial passes, one or both of the grafts displaces downwards or upwards; you just pass the next throw below, and when you put tension on the suture it will be aligned with the most dorsal border of the septum. When you reach the end (usually the keystone area), you can lock the suture, or more frequently, comeback caudally the running suture but this time including a bite of the upper lateral cartilages adhering them to the spreader grafts and septum. Beware this time not to put the suture below the spreader graft as in the beginning, but to take all 5 cartilages at the same level so the spreader graft effect (widening of the internal nasal valve or septum dorsum) is not lost (Fig. 1). Also, none of the bites includes the mucosa, so all the fixation is done with the cartilages. In this manner, no extrusion happens. At the end of the comeback suture, you can secure it knotting with the starting suture tail. Also, it is an excellent choice to perform the starting suture in a way the knot be inside the cartilages and will not be visible or palpable.

## RESULTS

We have performed this suture in 100 open rhinoplasty patients in the last 5 years. The most common indication was an open roof, to help to align the dorsum or to widen the dorsum to restore the dorsal esthetic lines. The most common difficulty with this technique is passing the suture at the keystone area because sometimes the nasal bones are long, and you cannot suture them. In this situation, fixation is restrained, but the spreader graft is usually very well fixated and there is no need for doing anything else and start the comeback suture.

Some advantages we have found with this technique are short learning curve, strong fixation, stability, and symmetry but overall the simplicity.

Disadvantage is the knot at the beginning or end of the fixation. We have not had any patient complaints for feeling the suture in the dorsum, even in thin skin patients, but we recommend only throw 2–3 knots maximum, so does not become bulky. We followed the patients 5 years to 6 months, and none of the patients needed a revision related to the spreader grafts fixation technique.

## CONCLUSIONS

Performing rhinoplasty is one of the most challenging surgeries in plastic surgery. Sheen gave us an excellent tool describing the spreader grafts, and surgeons use it to fix the cartilages with horizontal mattress suture. But for the surgeons who find sometimes difficult to align or to fix the spreader grafts with mattress sutures, we share this technique, (a running suture), which we find secure, fast, and easy to perform.
